# Multisystemic presentation of disseminated renal cell carcinoma

**DOI:** 10.1093/ehjcr/ytae561

**Published:** 2024-10-21

**Authors:** Karim Jamhour-Chelh

**Affiliations:** Department of Cardiology, Hospital Universitari Sant Joan de Déu, Fundació Althaia, Manresa 08243, Spain

A 62-year-old man with no known previous cardiovascular history presented to the emergency department with a 3-month history of asthenia, anorexia, and subacute dyspnoea. He also reported right motor syndrome over the last 6 h. Signs of migratory thrombophlebitis were evident. High levels of D-dimer (1850 mg/L) and anaemia were observed with haemoglobin level of 8.2 g/dL.

Both the computed tomography (CT) scan and X-ray showed cannonball images (*Panel A*, top; Supplementary material online, *Video S1*). The magnetic resonance imaging (MRI) revealed a subacute lesion on the left thalamus (*Panel B*, asterisk). The POCUS protocol detected a mass occupying 75% of the right ventricle. Pulmonary thromboembolism (see Supplementary material online, *Video S1*) was ruled out. Computed tomography scan showed a large mass (*Panel A*, down and asterisk) infiltrating the apex. *Panel C* shows a large mass in the left kidney (double asterisk) infiltrating the spleen with significant splenomegaly (asterisk). Additionally, there were signs of renal vein thrombosis (see Supplementary material online, *Video S1*).

Cardiac MRI (*Panel D*) revealed a large mass infiltrating apex, interventricular septum, and right ventricle free wall. It exhibited a mild hypointense signal with the myocardium on the bright blood (asterisk), while showing a slight hyperintense signal in both T1 and T2 mapping images (*Panel D*, top middle and right). Furthermore, a heterogeneous gadolinium enhancement was observed (see Supplementary material online, *Video S1*), indicating the presence of numerous metastatic implants in the left ventricle (*Panel D*, bottom yellow arrows). The endomyocardial biopsy (*Panel E*) confirmed a bulky mass in the right ventricle with several metastatic implants to the left ventricle from a disseminated sarcomatoid clear cell renal carcinoma (*Panel E*, red arrows) with multiple thromboembolic events. Unfortunately, the patient died from multiple organ dysfunction syndrome a few weeks after diagnosis.

**Figure:**
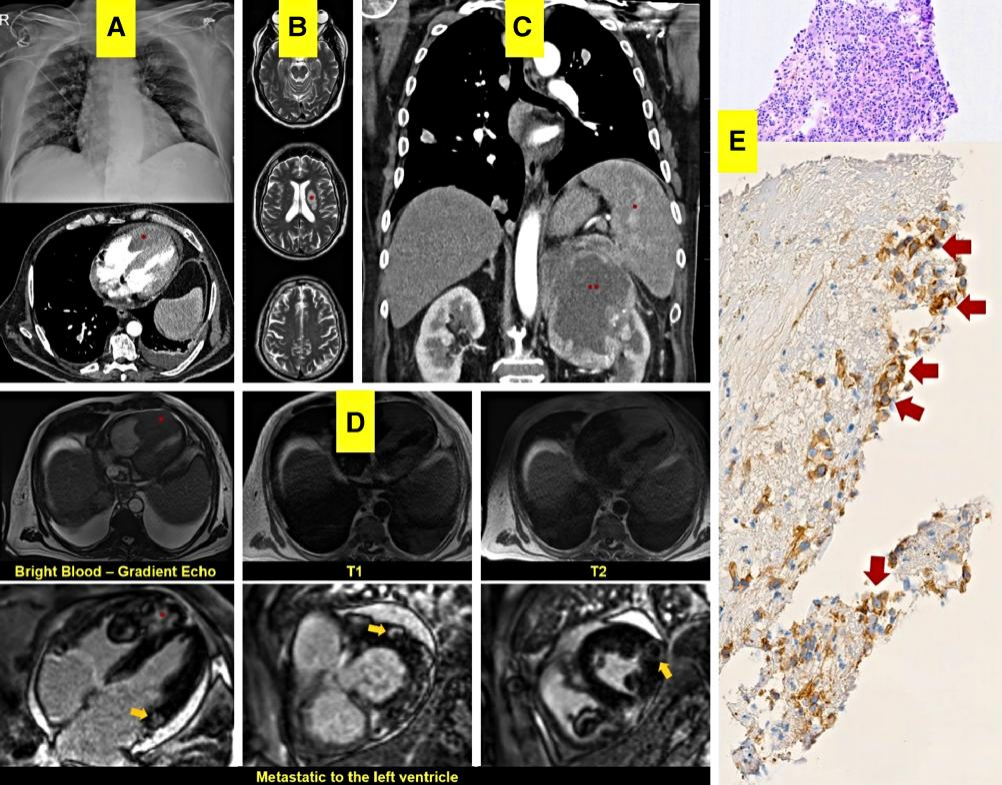
(*A*) Chest X-ray and computed tomography scan abnormalities. The image above is a clinical posterior–anterior chest X-ray image with radiologic signs of balloon loosening suggestive of pulmonary metathesis. The image below corresponds to a computed tomography scan at the thoracic level. After contrast administration, a mass invading 75% of the right ventricular cavity is observed (red marker), as well as the pleural effusion of left posterior predominance. (*B*) Cranial magnetic resonance imaging (MRI) scan without contrast. T2 MRI of the brain reveals high-signal-intensity large subacute lesion in the left-sided thalamus (red marker). (*C*) Thoracoabdominal computed tomography scan. A significant infiltrative mass is observed at the level of the upper pole of the left kidney, resulting in near-total distortion of the organ. This mass infiltrates adjacent structures (red markers), adhering to the left adrenal gland and spleen, leading to significant splenomegaly (red marker). Note the asymmetry between both hemidiaphragms. Additionally, multiple solid and atelectasis lesions, corresponding to multiple pulmonary metastases, are observed in the pulmonary field. On the left side of the image, a metastasis is infiltrating through the diaphragm. Additionally, irregularities in the hepatic surface suggest neoplasia formation. (*D*) Cardiac MRI scan. The upper images show axial steady-state free precession cardiac MRI image shows mass extension (red marker) into pericardium and obliteration of epicardial fat suspicious for myocardial invasion. Axial (down, left) and short-axis (down, middle, and right) myocardial delayed contrast enhancement cardiac MRI image shows a mass in both right and left ventricles with the focus on internal enhancement (red marker) and annular peripheral in all walls of the left ventricle (arrows) consistent with metastatic disease. (*E*) Histological features of endomyocardial mass biopsies. The slides show cells with clear cytoplasm. The distribution of the cells shows solid masses with a very abundant capillary stroma of spindle-shaped (sarcomatoid). On haematoxylin–eosin stained slides, there are areas of tumour with clear cell features and areas of papillary growth with more oncogenic characteristics. The immunohistochemical study revealed the presence of low molecular weight cytokeratins (CAM 5.2), vimentin, and 100 kDa surface metallopeptidase (CD10, arrows).

## Supplementary Material

ytae561_Supplementary_Data

## Data Availability

The data underlying this article will be shared on reasonable request to the corresponding author.

## Supplementary material

**Consent:** Since this was a routine case, the study did not require approval by the centre's Ethics Committee. Informed consent was obtained from the patient for the performance of the procedure and subsequent publication of the case.

**Funding:** There is no funding in this report.

